# Differential Age-Related Changes in Structural Covariance Networks of Human Anterior and Posterior Hippocampus

**DOI:** 10.3389/fphys.2018.00518

**Published:** 2018-05-09

**Authors:** Xinwei Li, Qiongling Li, Xuetong Wang, Deyu Li, Shuyu Li

**Affiliations:** ^1^School of Biological Science and Medical Engineering, Beihang University, Beijing, China; ^2^Beijing Advanced Innovation Centre for Biomedical Engineering, Beihang University, Beijing, China

**Keywords:** network, structural covariance, normal aging, anterior hippocampus, posterior hippocampus, MRI

## Abstract

The hippocampus plays an important role in memory function relying on information interaction between distributed brain areas. The hippocampus can be divided into the anterior and posterior sections with different structure and function along its long axis. The aim of this study is to investigate the effects of normal aging on the structural covariance of the anterior hippocampus (aHPC) and the posterior hippocampus (pHPC). In this study, 240 healthy subjects aged 18–89 years were selected and subdivided into young (18–23 years), middle-aged (30–58 years), and older (61–89 years) groups. The aHPC and pHPC was divided based on the location of uncal apex in the MNI space. Then, the structural covariance networks were constructed by examining their covariance in gray matter volumes with other brain regions. Finally, the influence of age on the structural covariance of these hippocampal sections was explored. We found that the aHPC and pHPC had different structural covariance patterns, but both of them were associated with the medial temporal lobe and insula. Moreover, both increased and decreased covariances were found with the aHPC but only increased covariance was found with the pHPC with age (*p* < 0.05, family-wise error corrected). These decreased connections occurred within the default mode network, while the increased connectivity mainly occurred in other memory systems that differ from the hippocampus. This study reveals different age-related influence on the structural networks of the aHPC and pHPC, providing an essential insight into the mechanisms of the hippocampus in normal aging.

## Introduction

With the population aging, understanding normal brain changes are as important as understanding demented diseases. Memory decline is a typical characteristic of normal aging. The hippocampus is considered critical in human memory and spatial navigation (Scoville and Milner, 1957; Buzsáki and Moser, 2013). Evidence suggests that hippocampal volume changes throughout the lifespan, which stays relatively stable until the age of 60 shows a sharp decline (Raz et al., 2010; Schuff et al., 2012; Fjell et al., 2013). Functional imaging studies have revealed and hypometabolism (de Leon et al., 2001; Wu et al., 2008) of the hippocampus in aging. Moreover, a reduced fractal dimension of hippocampal dynamics with age was reported (Goldberger et al., 2002; Wink et al., 2006).

The hippocampus differs in structure and function along its longitudinal axis (Poppenk et al., 2013). The anterior hippocampus (aHPC) and posterior hippocampus (pHPC) vary in pyramidal cell density (Babb et al., 1984; King et al., 2008) and have different developmental trajectories (DeMaster et al., 2014). Compared with young adults, both the aHPC and the pHPC showed volumetric atrophy in old adults (Pruessner et al., 2001; Chen et al., 2010; Rajah et al., 2010), and their rates of atrophy were different (Malykhin et al., 2008; Chen et al., 2010). Besides, an fMRI study reported the functional connectivity of the aHPC and pHPC were differentially affected in aging (Damoiseaux et al., 2016).

For structural connectivity, the structural covariance network (SCN) approach provides an effective way to characterize inter-regional structural covariance pattern of gray matter (GM) morphological properties (Mechelli et al., 2005; Modinos et al., 2009; Seeley et al., 2009; Zielinski et al., 2010; Montembeault et al., 2012; Li et al., 2013; DuPre and Spreng, 2017). The GM morphological covariance may result from direct white matter connection or neuronal co-activation (Alexander-Bloch et al., 2013). Studies have revealed a consistency among SCNs, anatomical connectivity networks, and functional connectivity networks, which provides strong support for using SCN mapping approach to assess network integrity. Age-related alteration of structural covariance in sensorimotor and cognitive networks has been found (Montembeault et al., 2012; Li et al., 2013). However, the effects of aging on the structural covariance of the aHPC and pHPC remain to be studied, which may provide insights into the hippocampal-related mechanism of aging and demented diseases.

In this study, we utilized a seed-based SCN approach to investigate the anterior and posterior hippocampal structural networks in 240 healthy subjects that were subdivided into young, middle-aged, and elderly groups. We first defined aHPC and pHPC based on the location of uncal apex in the MNI space. Then, we identified the SCNs seeding from aHPC and pHPC and compared the structural covariance differences between age groups. We expected the SCNs of the aHPC and pHPC have different patterns and were differently affected by age.

## Materials and Methods

### Participants

The MRI data were obtained from the publicly available Open Access Series of Imaging Studies (OASIS) database (Marcus et al., 2007). The OASIS database consists of 416 subjects aged 18–96, including 100 mild dementia and 316 healthy subjects. Based on the age distribution of the OASIS database, we selected 240 participants from the healthy subcohort and grouped them into young (18–23 years), middle-aged (30–58 years), and elderly (61–89 years) groups, with 80 participants in each group (see **Table 1**). All the subjects are right-handed and cognitively normal, with the Mini-Mental State Examination scores (Folstein et al., 1975) above 29 and the Clinical Dementia Rating scores (Folstein et al., 1975) equal zero. The same group of subjects was used in our previous study (Li et al., 2013).

**Table 1:** Participant characteristics by age group.

Group	Sample size (Females)	Age in years (mean ± SD)
Young	80 (50)	18–23 (20.66 ± 1.47)
Middle-aged	80 (50)	30–58 (47.43 ± 8.23)
Old	80 (55)	61–89 (73.75 ± 7.12)


### Data Acquisition

All MRI scans were performed on 1.5 Tesla Siemens scanners. For each individual, three to four T1-weighted images were acquired using a magnetization-prepared rapid gradient echo (MPRAGE) sequence with the following parameters: repetition time = 9.7 ms; echo time = 4 ms; inversion time = 20 ms; delay time = 200 ms; flip angle = 10°; matrix = 256 × 256; field of view = 256 mm; slices = 128; slice thickness = 1.25 mm. After motion corrected, the images of each subject were averaged to improve the contrast-to-noise ratio.

### Image Preprocessing

We used the VBM8 toolbox^1^ runs within SPM8 to implement voxel-based morphometry analysis (Ashburner and Friston, 2000) of the structural images. The acquired anatomical images were tissue classified into GM, white matter and cerebrospinal fluid images using tissue priors. Then, the segmented images were bias corrected and registered to a standard space using an affine transformation and a high-dimensional non-linear registration approach (Ashburner and Friston, 2005). Next, modulation of the segmented images was performed to correct for different individual brain size by using the non-linear registration parameters. Finally, the modulated GM segments were smoothed using an isotropic 12 mm full-width at half maximum Gaussian kernel for the structural covariance analysis.

### Definition of the Hippocampal Seeds

**FIGURE 1:**
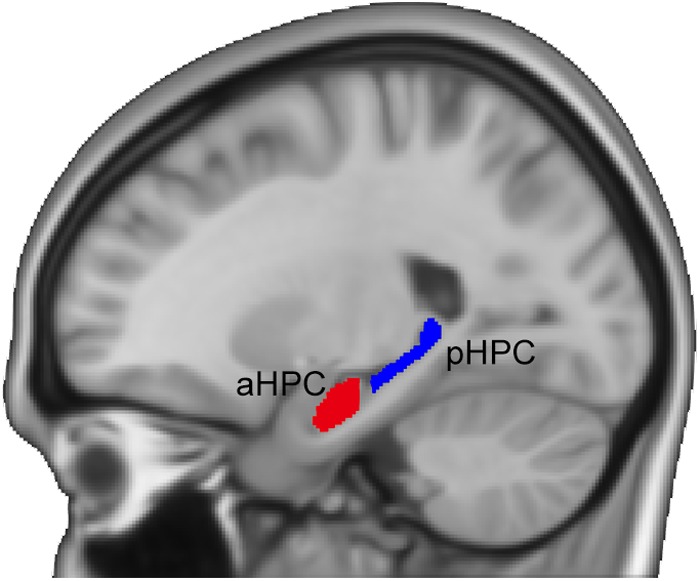
Illustration of the anterior and posterior hippocampal seeds. aHPC, anterior hippocampus; pHPC, posterior hippocampus.

Following previous studies (Poppenk et al., 2013; Persson et al., 2014), we adopted a MNI-coordinate-based segmentation method to partition the hippocampus. The hippocampus was identified using the Harvard-Oxford subcortical structural atlas (Desikan et al., 2006) from the FSL Software Library (Smith et al., 2004). Next, the left and right hippocampi were divided into the anterior and posterior sections separately based on the location of uncal apex in the MNI space (i.e., *Y* = -21 mm) (Poppenk et al., 2013). To avoid contamination effects between the aHPC and the pHPC, we removed a 2-mm coronal slice from each of the two adjacent ends (see **Figure 1**). For each subject, we measured the mean volumes of the hippocampal subfields from the modulated GM images using the MarsBar ROI toolbox^2^. Then a quadratic regression model was used to investigate age effects on the mean volumes of the anterior and posterior hippocampal segments. We also assessed the age-related hippocampal volumetric dispersion. To do so, for age = *t*, we calculated the variance of hippocampal volumes of subjects with age ∈ [*t*-2, *t*+2] and examined its quadratic relationship with age.

### Structural Covariance Analysis

Four separate regression analyses were executed on the modulated GM images data to map SCNs of the bilateral aHPC and pHPC in the young group. The model fitted the target voxel GM volume *Y* as:

$$Y∼\beta_{0}+\beta_{1}\left(Seed\right)+\beta_{2}\left(Gender\right)$$

where β_0_ is the intercept term, β_1_ model the relationship between the target voxel volume and the seed volume, and the Gender term was included as a nuisance variable. Total intracranial volume was not included because the modulation step already considered the brain size differences. These statistical analyses enable us to determine voxels that expressed a significant positive correlation with each seed. The criterion for significance was set at height and extent thresholds of *p* < 0.05, family-wise error (FWE) corrected for multiple comparisons. The resulting correlation maps were displayed on a standard brain template using the BrainNet Viewer (Xia et al., 2013) to allow qualitative comparisons the structural covariance patterns of hippocampal seeds.

We further assessed the influence of age on the regional structural covariance between the hippocampus and the rest brain regions by using a classic linear interaction model (Lerch et al., 2006). For any two age groups, the target voxel volume *Y* was modeled as follows:

$$Y∼\beta_{0}+\beta_{1}\left(Seed\right)+\beta_{2}\left(Group\right)+\beta_{3}(Gender)+\beta_{4}(Group\times Seed)$$

where β_0_ is the intercept term, β_1_ ∼ β_4_ models the relationship between the target voxel volume and the seed volume, group term, gender term, and interaction term (group by seed), respectively. To obtain between-group differences, specific *t* contrasts were established to test the statistical significance of the interaction term. Clusters with height and extent thresholds set at *p* < 0.05 (FWE corrected) were considered significant.

## Results

### Hippocampal Volume Analyses

**FIGURE 2:**
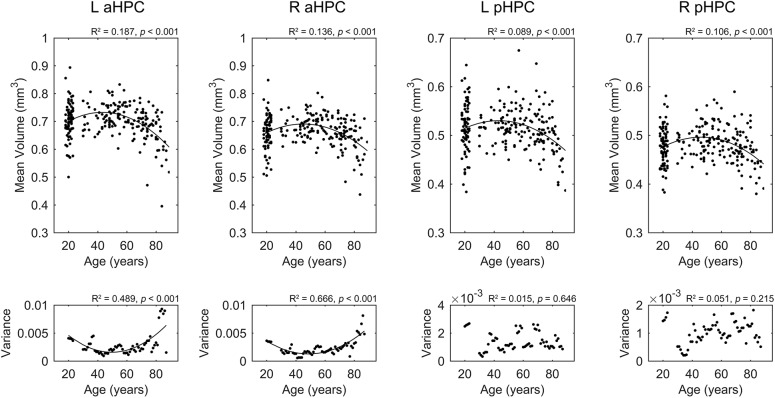
Life-span trajectories of the anterior and posterior hippocampal mean gray matter volumes. The lower row shows the relationship between age and the variance of hippocampal volumes in a small age range. aHPC, anterior hippocampus; pHPC, posterior hippocampus; L, left, R, right.

Results for the regression analysis of anterior and posterior hippocampal mean GM volumes versus age are presented in **Figure 2**. Similar nonlinear relationship between the bilateral hippocampal volumes and age were found: the volumes slightly increased before the age of 50 and then decreased sharply (left aHPC: *R*^2^ = 0.187, *p* < 0.001; right aHPC: *R*^2^ = 0.136, *p* < 0.001; left pHPC: *R*^2^ = 0.089, *p* < 0.001; right pHPC: *R*^2^ = 0.106, *p* < 0.001). Moreover, the results suggested that the mean volume of the aHPC was larger than the pHPC, and the left hippocampal volume was slightly greater than the right side. In addition, we found the variance of the bilateral anterior hippocampal volumes has an age-related U-shaped relationship (left aHPC: *R*^2^ = 0.489, *p* < 0.001; right aHPC: *R*^2^ = 0.666, *p* < 0.001). Specifically, the anterior hippocampal volumes of the young and old subjects were more dispersed than the middle age. However, the variance of the posterior hippocampal volumes did not significantly relate to age (left pHPC: *R*^2^ = 0.015, *p* = 0.646; right pHPC: *R*^2^ = 0.051, *p* = 0.215).

### Structural Covariance Networks of the Anterior and Posterior Hippocampus

**FIGURE 3:**
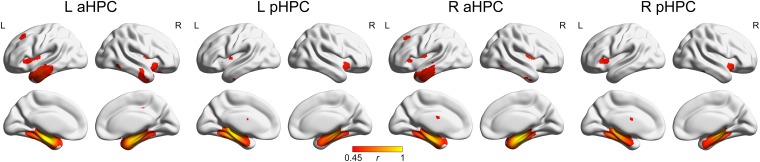
Structural covariance networks of the anterior and posterior hippocampus in the Young group. Regions with *P*_FWE_ < 0.05 are presented as correlation coefficient values. aHPC, anterior hippocampus; pHPC, posterior hippocampus; L, left; R, right.

The SCNs seeding from the aHPC and pHPC in the young participants are presented in **Figure 3** (*p* < 0.05, FWE corrected). The aHPC correlated with the bilateral temporal lobe (including the superior, middle and inferior temporal, parahippocampal gryi, entorhinal cortex, fusiform and temporal pole), amygdalae, insula and posterior cingulate gyrus, orbitofrontal cortex, as well as left superior frontal gyrus. For the pHPC, its covariance maps involved the bilateral medial temporal regions (including the parahippocampal gyrus, entorhinal cortex and fusiform), amygdalae and insula. Noted that the regions correlated with both the aHPC and pHPC were mainly located in the medial temporal lobe and insula.

### Age-Related Differences Within the Anterior Hippocampal Network

**FIGURE 4:**
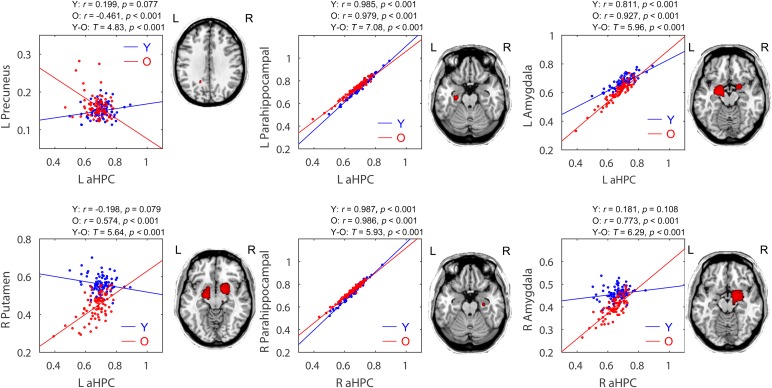
Age-related group differences in structural covariance of the anterior hippocampus. Correlations between the mean volume of the anterior hippocampus and the regional gray matter volumes extracted from a 4-mm-radius sphere centered on the peak voxel of a significant cluster (*P*_FWE_ < 0.05, shown on the right) are displayed. Y, young group; O, old group; L, left; R, right.

Within the anterior hippocampal network, significant between-group differences were only observed between the young group and the old group (*p* < 0.05, FWE corrected, **Figure 4** and **Table 2**). Specifically, the left and right aHPC showed decreased positive correlation with the ipsilateral parahippocampus and increased positive correlation with the ipsilateral amygdala in the old group relative to the young group. Moreover, compared to the young group, the left aHPC exhibited lower structural covariance with the left precuneus and greater structural covariance with the right putamen in the old group.

**Table 2:** Significant between-group differences in structural association between hippocampal seeds and other anatomical regions.

Seed	Contrast	Anatomical region	MNI coordinates			Cluster size	Max*T*
					
			*X*	*Y*	*Z*		
L aHPC	Y > O	L Parahippocampus	-27	-16	-21	126	7.08
		L Precuneus	-18	-52	31	18	4.83
	Y < O	L Amygdala	-21	-7	-15	1314	5.96
		R Putamen	18	3	-11	1096	5.64
R aHPC	Y > O	R Parahippocampus	27	-15	-21	36	5.93
	Y < O	R Amygdala	16	-1	-14	1871	6.29
L pHPC	Y < O	R Caudate	12	10	-6	5	4.51
R pHPC	M < O	R Temporal pole	62	0	-17	29	4.70
	Y < O	R Putamen	14	8	-6	1655	5.42
		L Putamen	-22	3	-3	1084	5.04


**P* < 0.05, FWE corrected. Abbreviations: L, left; R, right; aHPC, anterior hippocampus; pHPC, posterior hippocampus; Y, young group; M, middle-aged group; O, old group.*

### Age-Related Differences Within the Posterior Hippocampal Network

**FIGURE 5:**
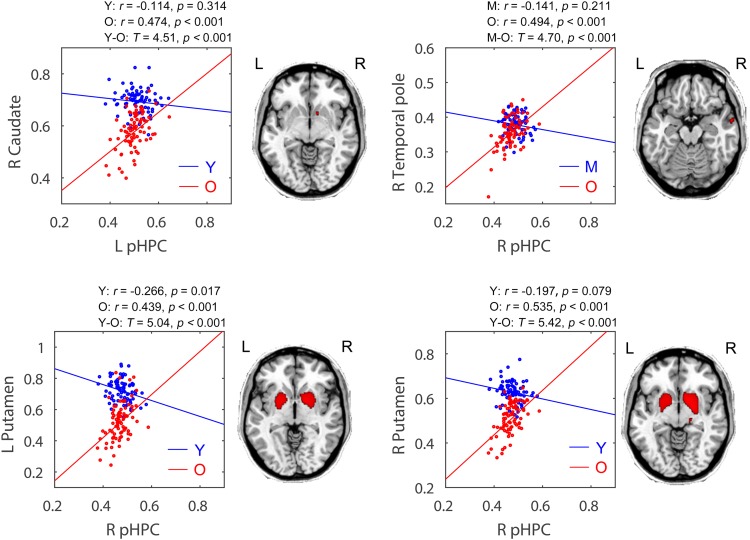
Age-related group differences in structural covariance of the posterior hippocampus. Correlations between the mean volume of the posterior hippocampus and the regional gray matter volumes extracted from a 4-mm-radius sphere centered on the peak voxel of a significant cluster (*P*_FWE_ < 0.05, shown on the right) are displayed. aHPC, anterior hippocampus; pHPC, posterior hippocampus; Y, young group; M, middle-aged group; O, old group; L, left; R, right.

Within the posterior hippocampal network, only increased structural associations were found in the old group relative to younger adults (mainly the young group, *p* < 0.05, FWE corrected, see **Figure 5** and **Table 2**). For the left pHPC, the old group exhibited significantly increased connectivity with the right caudate related to the young group. For the right pHPC, its connection with bilateral putamen was negative in the young group but was positive in the old group. Similarly, the right pHPC and temporal pole was negatively related in the middle-aged group but positively related in the old group.

## Discussion

Here, we studied the age-related structural covariance alterations of the aHPC and pHPC using a seed-based SCN approach. We found that the SCNs seeding from the aHPC and pHPC in the young adults were different from each other, but both of them related with the medial temporal lobe and insula. In addition, the structural covariance differences within the anterior hippocampal network were mainly between the young group and the old group with both decreased and increased positive structural associations. While compared to the younger adults, only increased structural associations were found in the old group within the posterior hippocampal network.

We observed that the volumes of aHPC/pHPC slightly increased from young to middle age, and then decreased sharply with age. In line with this finding, several morphometric studies reported an inverted U pattern of the hippocampal volume changes with age (Walhovd et al., 2005; Li et al., 2014). As the hippocampus is important in memory processing, this pattern may partially explain the similar age-related memory change trajectory (Nyberg et al., 2012). Interestingly, we found that the anterior hippocampal volumes of the young and old subjects are more dispersed than the middle age, may pointing to stronger heterogeneity memory ability in young and old subjects. Whether this age-related dispersion due to the sample selection or other reasons requires further analysis.

Structural covariance analyses suggested that the aHPC connected with temporal lobe, amygdala, insula, and orbitofrontal cortex (*p* < 0.05, FWE corrected), which agree with previous studies (Kier et al., 2004; Smith et al., 2009; Catenoix et al., 2011). And the pHPC was covariant with medial temporal amygdala, and insula (*p* < 0.05, FWE corrected) showing consistent connections with previous studies by using fMRI and tractography (Kahn et al., 2008; Poppenk and Moscovitch, 2011; Poppenk et al., 2013). The common related regions with both aHPC and pHPC were mainly located in the medial temporal lobe where the hippocampus located.

Age-related decrements in structural covariance were observed in the aHPC-related SCNs (*p* < 0.05, FWE corrected). In particular, the parahippocampal gyrus and precuneus showed reduced association with the aHPC seed in old adults relative to young adults. The parahippocampal gyrus is considered as a mediator between the cortical DMN subsystem and the hippocampus (Ward et al., 2014), and the integrity of the cortico-parahippocampus-hippocampus circuit is important for learning and episodic memory (Witter et al., 2000; Van Strien et al., 2009). Therefore, the weakened parahippocampus-hippocampus connection may lead to memory deficits in normal elderly, and result in decreased structural covariance between the hippocampus and cortical regions, such as the precuneus found in this study. Besides, the decreased connectivity between the precuneus and hippocampus might result from very early beta-amyloid deposition of the precuneus in elderly subjects (Sheline et al., 2010). The abnormal synaptic activity caused by amyloid deposition might disrupt cortico-hippocampal connectivity, which then results in hippocampal atrophy (Mormino et al., 2009).

Note that the parahippocampal gyrus, precuneus, and hippocampus are all components of DMN (Andrews-Hanna et al., 2014). Thus, our findings may indicate that aging is associated with decreased structural covariance within the DMN, which is in keeping with observations from previous SCN studies (Montembeault et al., 2012; Li et al., 2013; Spreng and Turner, 2013). A previous study reported decreased fractal complexity in DMN with age using multifractal analysis of fMRI series (Ni et al., 2014). Moreover, aging-related decrements in functional connectivity (Damoiseaux et al., 2008; Tomasi and Volkow, 2012) and white matter integrity (Damoiseaux et al., 2009; Brown et al., 2015) of DMN were also reported. Since DMN is known to play a role in episodic memory processing (Greicius et al., 2004, 2009), its decreased integrity could underlie memory impairment in senior populations (Salami et al., 2014).

Additionally, our data suggest that the influence of age on the structural connectivity between the hippocampus and cortical DMN nodes may be limited to the anterior portion of the hippocampus. Similarly, Salami et al. (2014) revealed reduced functional connectivity between the cortical DMN subsystems and more anteriorly located hippocampus with advancing age. Several fMRI studies have demonstrated the aHPC as part of DMN was engaged in episodic memory (autobiographical memory) processing (Zeidman and Maguire, 2016). However, some studies found no age-related differences for the connectivity between the aHPC and DMN regions (Koch et al., 2010; Damoiseaux et al., 2016), while others reported lower connectivity between the pHPC and DMN regions in older adults (Andrews-Hanna et al., 2007; Damoiseaux et al., 2016). These discrepancies may be due to methodological differences, notably in the type of measurements and sample characteristics, which should be further investigated.

Moreover, age-related increments in structural covariance were observed in both the aHPC- and pHPC-related SCNs (*p* < 0.05, FWE corrected). Particularly, compared to young adults, the putamen and amygdala showed increased associations within the aHPC-related SCNs in old adults. Within the pHPC-related SCNs, the putamen, caudate, and temporal pole showed increased associations in old adults relative to younger adults. The putamen and caudate form the dorsal striatum. In fact, the hippocampus, dorsal striatum, and amygdala belong to different memory systems and play different roles in information acquisition (McDonald and White, 1993). The dorsal striatum and hippocampus cooperate to support episodic memory function (Sadeh et al., 2011), while the amygdala plays a role in regulating these two memory systems (Packard and Teather, 1998). We speculated that the age-related increment in hippocampal structural covariance may reflect the compensatory mechanism or dedifferentiation effects of the brain memory systems during aging (Dennis and Cabeza, 2011; Oedekoven et al., 2015).

The greater structural covariance between the hippocampus and dorsal striatum (caudate-putamen) in older adults may also be related to non-optimal dopamine processing. The CA1 area of the hippocampus receives dopaminergic modulation from the ventral tegmental area, which plays a vital role in synaptic plasticity of the hippocampus (Lisman and Grace, 2005). But the ventral tegmental area suffers from dopamine neurons loss (Siddiqi et al., 1999) and reduced dopamine transporter function (Salvatore et al., 2003) with age. However, the dorsal striatum, another area in the dopamine system, increases its dopamine synthesis capacity in aging (Braskie et al., 2008). Thus, the increased connections between the hippocampus and dorsal striatum during aging suggest compensation for deficits in the ventral tegmental area, which may represent non-optimal dopamine system functioning.

The SCN method used in this study provides an effective way to construct brain networks from medical images, which complements the signal analysis methods (Liu et al., 2015). However, since aging is not only characterized by brain deficits but also decline in multiple organ functions, it is interesting to utilize the integrative approaches within the new filed of network physiology to study the effects of aging on brain–brain or brain–organ networks in future (Bashan et al., 2012; Bartsch et al., 2015; Ivanov et al., 2016). In addition, it is worth noting that brain networks have a fractal property of hierarchical modularity, which confers robustness of network function (Bullmore and Sporns, 2012). Future studies using fractal analysis approaches (Meunier et al., 2010; Xue and Bogdan, 2017) to study the complexity and heterogeneity of hippocampal networks could advance our understanding of the brain in normal aging.

## Author Contributions

XL designed and performed the experiments, analyzed the data, and drafted the manuscript. QL and XW helped to analyze the data and to draft the manuscript. DL and SL contributed to the study design, coordination, and final approval of the manuscript. All authors read and approved this version to be published.

## Conflict of Interest Statement

The authors declare that the research was conducted in the absence of any commercial or financial relationships that could be construed as a potential conflict of interest.

## Footnotes

1 http://www.neuro.uni-jena.de/vbm/

2 http://marsbar.sourceforge.net/
